# Turn-On Fluorescent Chemosensor for Hg^2+^ Based on Multivalent Rhodamine Ligands

**DOI:** 10.3390/ijms131216822

**Published:** 2012-12-07

**Authors:** Xuemei Wang, Mudassir Iqbal, Jurriaan Huskens, Willem Verboom

**Affiliations:** 1 Laboratory of Molecular Nanofabrication, MESA+ Institute for Nanotechnology, University of Twente, Enschede 7500 AE, The Netherlands; E-Mails: wxm_julia@163.com (X.W.); m.iqbal@utwente.nl (M.I.); j.huskens@utwente.nl (J.H.); 2 Department of Chemistry and Material Engineering, Logistic Engineering University, Chongqing 401311, China; 3 Department of Chemistry, University of Sargodha, Punjab 40100, Pakistan

**Keywords:** rhodamine, mercury, chemosensor, fluorescence

## Abstract

Rhodamine-based fluorescent chemosensors **1** and **2** exhibit selective fluorescence enhancement to Fe^3+^ and Hg^2+^ over other metal ions at 580 nm in CH_3_CN/H_2_O (3/1, *v*/*v*) solution. Bis(rhodamine) chemosensor **1**, under optimized conditions (CH_3_CN/HEPES buffer (0.02 M, pH = 7.0) (95/5, *v*/*v*)), shows a high selectivity and sensitivity to Hg^2+^, with a linear working range of 0–50 μM, a wide pH span of 4–10, and a detection limit of 0.4 μM Hg^2+^.

## 1. Introduction

Mercury is considered to be a highly dangerous element by the United States Environmental Protection Agency due to its special properties, such as migration through cell membranes and bioaccumulation within living tissues [1,2]. Therefore, there is a high demand for the determination of the Hg^2+^ ion in environmental analysis.

In recent years, rhodamine-based fluorescent chemosensors have received considerable attention for the detection of Hg^2+^[3–14], Cu^2+^[15–17], Pb^2+^[18], Cr^3+^[19], and Fe^3+^[20], because their special structural properties provide an ideal mode to construct off-on fluorescent switch chemosensors. Rhodamine having a spirolactam structure is non-fluorescent, whereas ring-opening of the spirolactam gives rise to a strong fluorescence emission. Moreover, they have a longer emission wavelength (about 550 nm), which is often preferred to serve as reporting group for analytes to avoid the influence of the background fluorescence (below 500 nm) [21–23]. However, most of them have shortcomings in practical application, such as cross-sensitivities toward other metal cations, low water solubility, a narrow pH span, and delayed response, *etc*. Accordingly, quantitative practical Hg^2+^ detection requires a linear fluorescence response, uniform fluorescence output at a broad pH range, compatibility with aqueous medium, high selectivity, sensitivity, and a fast response, while easy synthetic procedures for the sensors are of utmost importance.

**Chart 1:**
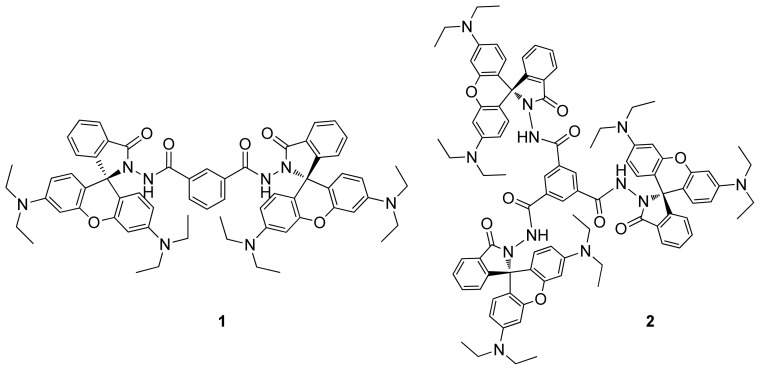
Structure of chemosensors **1** and **2**.

This study deals with new rhodamine-based CHEF (chelation-enhanced fluorescence) chemosensors **1** and **2** (Chart 1) for the detection of Hg^2+^ ions showing that, compared to related rhodamine-based chemosensors, small structural changes give rise to improved selectivity and sensitivity. Chemosensor **1** is a bis(rhodamine) in which the two units are connected via amide groups meta substituted to a benzene ring. In order to study the influence of a third functionalized rhodamine on the Hg^2+^ complexation, tris(rhodamine) chemosensor **2** was prepared and evaluated for comparison.

## 2. Results and Discussion

**Scheme 1:**
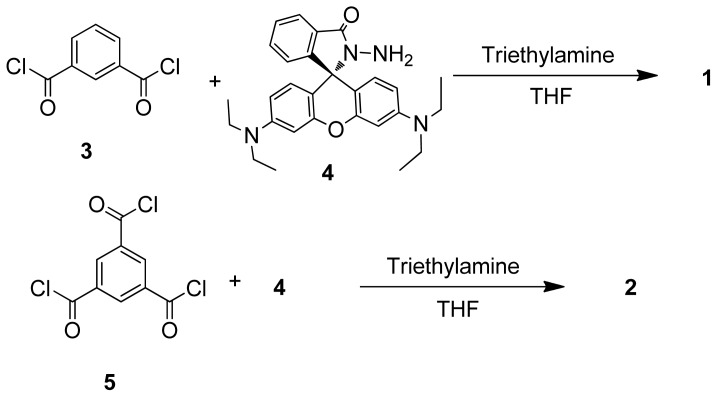
Synthesis of chemosensors **1** and **2**.

Rhodamine derivatives **1** and **2**, possessing two or three rhodamine moieties, respectively, were prepared by reacting rhodamine B hydrazide (**4**) with isophthaloyl dichloride (**3**) or benzene-1,3,5-tricarbonyl trichloride (**5**) in THF as a solvent (Scheme 1). The formation of **1** and **2** followed from the ^1^H NMR spectra as the doublets at 6.42 and 6.46 ppm in rhodamine B hydrazide (**4**) shifted to 6.61–6.75 ppm and 6.59–6.76 ppm as multiplets for **1** and **2**, respectively. In the ESI-MS mass spectra the [M+H] peaks were found at *m/z* 1043.5 and 1525.7 for **1** and **2**, respectively.

**Figure 1:**
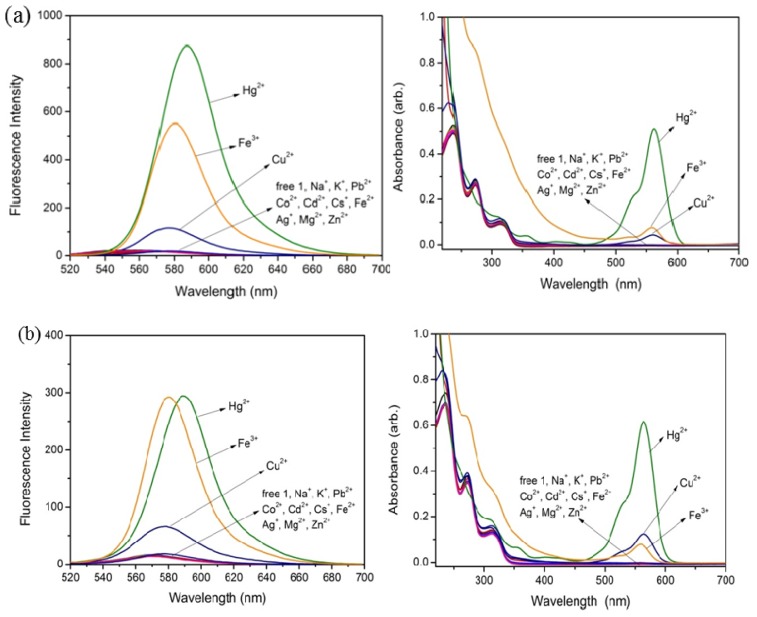
Fluorescence (left) and absorption (right) spectra of **1** (**a**) and **2** (**b**) (5 μM) in CH_3_CN/H_2_O (3/1, *v*/*v*) with different metal ions (400 μM), respectively.

**Figure 2:**
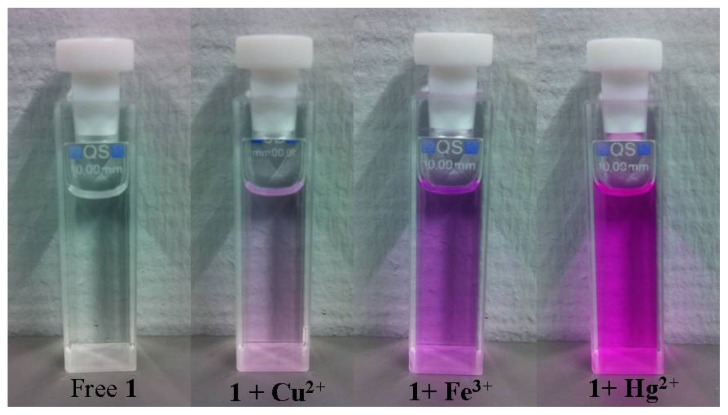
Photos of chemosensor **1** (5 μM) in CH_3_CN/H_2_O (3/1, *v*/*v*) upon addition of 80 equiv of Cu^2+^, Fe^3+^, and Hg^2+^ ions, respectively.

The perchlorate salts of Na^+^, K^+^, Pb^2+^, Co^2+^, Cd^2+^, Cs^+^, Ag^+^, Cu^2+^, Mg^2+^, Zn^2+^, Hg^2+^, Fe^2+^, and Fe^3+^ ions were used to evaluate the metal ion binding properties of chemosensors **1** and **2** in CH_3_CN/H_2_O (3/1, *v*/*v*). The fluorescence spectra were obtained by excitation of the rhodamine fluorophore at 510 nm. Among these metal ions (80 equiv), chemosensors **1** and **2** both showed large chelation enhanced fluorescence (CHEF) effects with Hg^2+^, Fe^3+^ and smaller CHEF effects with Cu^2+^ (Figure 1). The addition of 400 μM (80 equiv) of Fe^3+^ and Hg^2+^ immediately yielded a pink solution with a absorption signal at 561 nm [24] and a strong fluorescence signal at 580 and 590 nm, respectively (Figure 2). For chemosensor **1**, there was 35-fold enhancement with Fe^3+^ and 84-fold enhancement with Hg^2+^, while chemosensor **2** yielded a 27-fold enhancement with Fe^3+^ and 33-fold with Hg^2+^. The results can be attributed to a similar binding behavior of **1** and **2** both containing rhodamine moieties. In addition, a very weak fluorescence signal for free **1** and **2** was observed at 580 and 590 nm, respectively, upon excitation at 510 nm, confirming the presence of a ring-closed spirolactam structure, whereas with the addition of Fe^3+^ or Hg^2+^ ions, ring-opening of the spirolactam occurs and gives rise to a strong fluorescence emission at 580 and 590 nm, respectively. Though Cu^2+^ gave a small color change and a very small fluorescence enhancement, the spectroscopy and interaction of chemosensors **1** and **2** with Cu^2+^ are completely different from those of **1** and **2** with Fe^3+^ and Hg^2+^ as recently reported by others [20].

**Figure 3:**
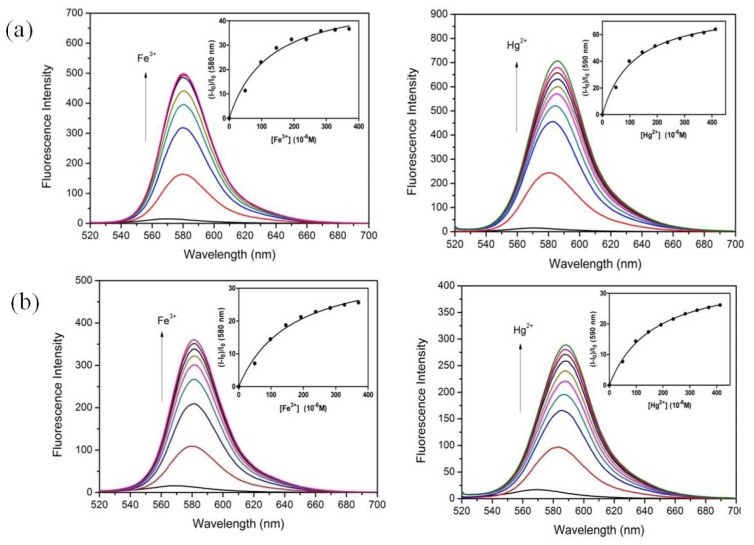
Changes of the fluorescence spectra of (**a**) chemosensor 1 and (**b**) chemosensor 2 (5 μM, λ_ex_ = 510 nm) in CH_3_CN/H_2_O (3/1, *v*/*v*) upon addition of increasing amounts of Fe^3+^ (0–370 μM, left) and Hg^2+^ (0–400 μM, right), respectively. Inset: Spectrofluorimetric titration curves ((**a**) λ_em_ =580 nm and (**b**) λ_em_ = 590 nm) for a 1:1 complex according to Equation 1. The data are fitted to a curve with a correlation coefficient of (**a**) *R*^2^ = 0.9952, *R*^2^ = 0.9972 and (**b**) *R*^2^ = 0.9945, *R*^2^ = 0.9983.

To investigate the binding mode and the affinity, Job’s plots (see Figure S1) were determined and fluorescence titration experiments were carried out for chemosensors **1** and **2** with Fe^3+^ and Hg^2+^ (Figure 3). The Job’s plots show that in all cases 1:1 complexes were formed. The resulting titrations also fitted to a 1:1 binding model, and the association constant (*K*_s_) can be gained using Equation 1 [25,26].

(1) $$\frac{\left(I-I_{0}\right)}{I_{0}}=\frac{\alpha \times [\text{M}]}{\left(1/K_{s})+[\text{M}\right]}$$

Where *I*_0_ is the fluorescence intensity of the chemosensors **1** and **2** in the absence of metal ions and *I* is the fluorescence intensity upon the addition of metal ions. α is the maximum specific binding, [M] is the metal ion concentration, *K*_s_ is the association constant.

The association constants for **1** with Fe^3+^ and Hg^2+^ were found to be 7.99 × 10^3^ M^−1^ and 8.62 × 10^3^ M^−1^, while those for **2** with Fe^3+^ and Hg^2+^ were 6.18 × 10^3^ M^−1^ and 6.08 × 10^3^ M^−1^, respectively, which are close to those of a related bis(rhodamine) chemosensor [20]. The *K*_s_ values of chemosensors **1** and **2** only marginally differ, those of tris(rhodamine) **2** even being slightly smaller than those of bis(rhodamine) **1**. In addition to the Job’s plot determination, this also demonstrates that two rhodamines are sufficient for optimal metal ion binding. In chemosensor **1**, two carbonyl oxygens as well as two amide oxygens can provide a stable binding pocket for metal ions.

**Figure 4:**
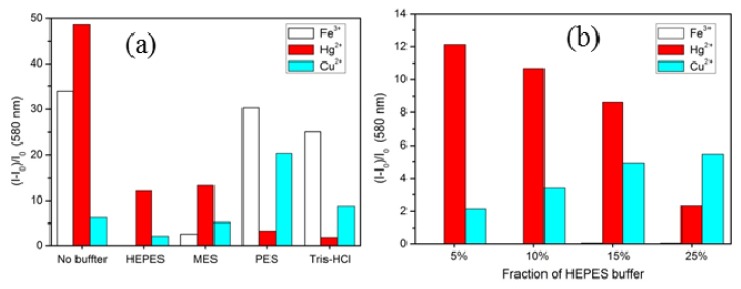
Fluorescence response of chemosensor 1 (5.0 μM, λ_ex_ = 510 nm and λ_em_ = 580 nm) (**a**) in CH_3_CN/buffer (pH = 7.0) (95/5, *v*/*v*) upon addition of 40 equiv of Fe^3+^, Hg^2+^, and Cu^2+^ ions, respectively, and (**b**) in different fractions of HEPES buffer (0.02 M, pH = 7.0) upon addition of 40 equiv of Fe^3+^, Hg^2+^, and Cu^2+^ ions, respectively. The responses for Fe^3+^ are below 0.05 in the different fractions of HEPES buffer.

To obtain a high selectivity and sensitivity for Hg^2+^ under aqueous conditions, HEPES buffer (0.02 M, pH = 7.0), MES buffer (0.01 M, pH = 7.0), PES buffer (0.01 M, pH = 7.0), and Tris HCl buffer (0.01 M, pH = 7.0) were used, respectively (Figure 4a). The fluorescence intensities upon addition of 40 equiv of Fe^3+^, Hg^2+^, and Cu^2+^ ions show the effect of the different buffer systems. In this case, HEPES effectively inhibits the interference of Fe^3+^ and Cu^2+^ ions during the detection of Hg^2+^.

The fraction of HEPES buffer used in CH_3_CN played an important role in the affinity of **1** toward Hg^2+^. Because of the strong interaction between buffer anions and Fe^3+^, a small fraction of HEPES buffer in organic solvent was already beneficial to inhibit the binding of Fe^3+^. However, a high fraction of HEPES buffer caused a decrease of the fluorescence emission for **1**·Hg^2+^ and an increase of that of the complex with Cu^2+^, which has to be avoided for Hg^2+^ analysis. To determine the optimal analysis condition, 5 μM chemosensor **1** in CH_3_CN containing different fractions (5%, 10%, 15%, and 25% (*v*/*v*)) of 0.02 M HEPES buffer at pH 7.0 were used for the detection of Fe^3+^, Hg^2+^, and Cu^2+^ (Figure 4b). No significant fluorescence enhancement could be observed at 580 nm for Fe^3+^ compared to that of Hg^2+^ and Cu^2+^ at the same concentration. The results already show that 5% HEPES/CH_3_CN was already sufficient for efficient monitoring of Hg^2+^.

**Figure 5:**
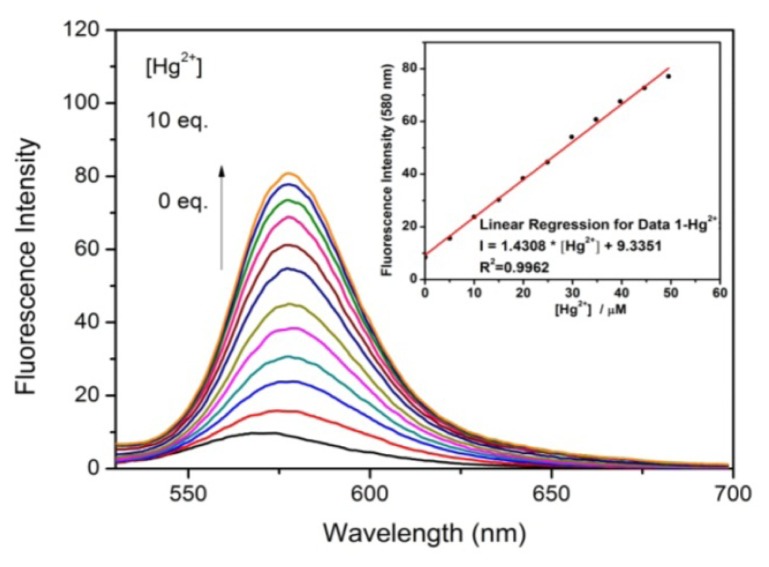
Changes of the fluorescence spectra of chemosensor **1** (5 μM, λ_ex_ = 510 nm) in CH_3_CN/HEPES buffer (0.02 M, pH = 7.0) (95/5, *v*/*v*) upon addition of increasing amounts of Hg^2+^ (0–50 μM). Inset: Fluorescence intensity of **1** at 580 nm (5 μM, λ_ex_ = 510 nm) in CH_3_CN/HEPES buffer (0.02 M, pH = 7.0) (95/5, *v*/*v*) *vs* the concentration of Hg^2+^ ions.

**Figure 6:**
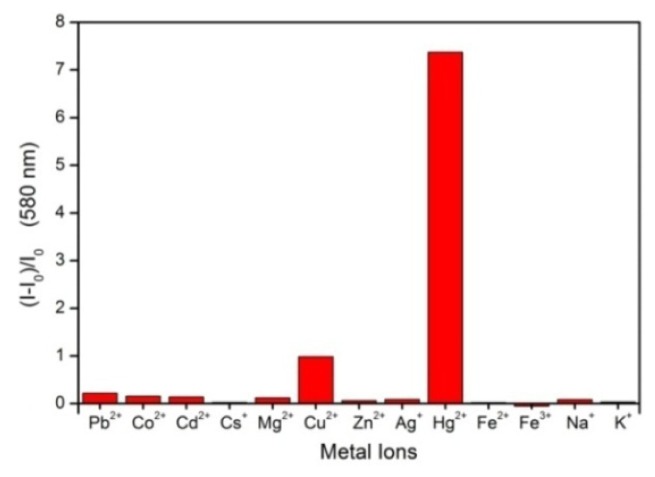
Bar profiles of fluorescence intensity for chemosensor **1** (5 μM, λ_ex_ = 510 nm) in CH_3_CN/HEPES (95/5, *v*/*v*) upon addition of 10 equiv of various metal ions as perchlorates.

Fluorescence titrations of Hg^2+^ by **1** were performed, under the optimized conditions of CH_3_CN/HEPES buffer (0.02 M, pH = 7.0) (95/5, *v*/*v*) (Figure 5). For the quantitative detection of Hg^2+^ ions, under the optimized conditions, a calibration curve was generated by determining the fluorescence intensity of **1** (5 μM) at 580 nm upon addition of Hg^2+^ ions with different concentrations, ranging from 0 to 50 μM [27]. Figure 5 exhibits over the entire Hg^2+^ concentration range an almost perfect linearity (*I*_580_ = 1.43 × [Hg^2+^] + 9.34, *R*^2^ = 0.9962) between the fluorescence intensity of **1** and the Hg^2+^ concentration, indicating a linear detection range for Hg^2+^ determination. The detection limit, defined as three times the standard deviation of the blank signals [28], was found to be 0.4 μM from 10 blank solutions. In addition, Figure 6 shows that chemosensor **1** detected Hg^2+^ ions with high selectivity under these conditions.

**Figure 7:**
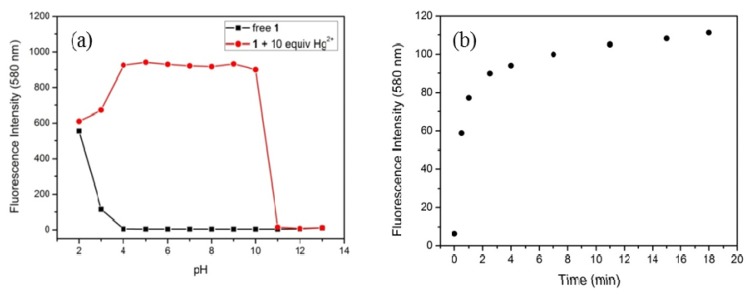
Fluorescence intensity of **1** at 580 nm (5 μM, λ_ex_ = 510 nm) in CH_3_CN/H_2_O (95/5, *v*/*v*) (**a**) with and without Hg^2+^ ion (50 μM) as a function of pH and (**b**) upon addition of Hg^2+^ ion (50 μM) over time.

For practical applicability of this new chemosensor, a proper pH range of 4–10 was determined. Figure 7a shows variations of the fluorescence intensity of **1** with pH in the absence and presence of the Hg^2+^ ion in CH_3_CN/H_2_O solution (95/5, *v*/*v*). In this region, free **1** has a weak fluorescence emission due to the presence of the ring-closed spirolactam structure, while addition of the Hg^2+^ ion leads to ring-opening of the spirolactam ring, resulting in a remarkable increase of the fluorescence.

The ring-opening of the spirolactam in chemosensor **1** produced a time-dependent dosimetric response, controlled by the reaction kinetics. Under the optimized conditions less than 4 min were required to complete the reaction (Figure 7b).

Chemosensor **1** in CH_3_CN/HEPES buffer (0.02 M, pH = 7.0) (95/5, *v*/*v*) has a low detection limit (0.4 μM), a large linear detection range (0–50 μM), a wide pH span (4–10), and a rapid response time (4 min), exhibiting higher sensitivity and selectivity than most other previously reported rhodamine-based chemosensors [3–14].

## 3. Experimental Section

### 3.1. General

Absolute acetonitrile of analytical grade and deionized water were used throughout the experiments. All chemicals needed for the synthesis were purchased from known suppliers and used without further purification. The known rhodamine B hydrazide (**4**) was prepared according to a literature procedure [29]. The metal ion solutions were prepared from their analytical grade perchlorate salts. HEPES buffer, MES buffer, PES buffer, Tris HCl buffer solutions and different pH solutions were prepared using proper amounts of HEPES, MES, PES, Tris, 1.0 M HCl, and 1.0 M NaOH (all of analytical grade) under adjustment by a pH meter.

### 3.2. Equipment

Absorption spectra were determined on a Perkin Elmer Lambda 850 UV-vis spectrophotometer. Fluorescence spectroscopy measurements were performed on a Perkin Elmer LS55 spectrofluorimeter equipped with a xenon discharge lamp and using 1 cm quartz cells. All pH measurements were made with a Mettler Toledo SevenEasy pH meter. ^1^H NMR and ^13^C NMR spectra were recorded on a Varian Unity INOVA (300 MHz) spectrometer in CDCl_3_. ^1^H NMR (300 MHz) and ^13^C NMR (75 MHz) chemical shift values are reported as δ using the residual solvent signal as an internal standard. Electrospray Ionization (positive mode) mass spectra were recorded on a WATERS LCT mass spectrometer.

### 3.3. Synthesis of **1** and **2**

#### 3.3.1. General Procedure for the Synthesis of **1** and **2**

To a solution of rhodamine B hydrazide (**4**) and triethylamine in THF a solution of isophthaloyl dichloride (**3**) or benzene-1,3,5-tricarbonyl trichloride (**5**) in THF was added dropwise at 0 °C. The reaction mixture was brought to room temperature in 1 h, followed by stirring overnight at room temperature. The solvent was evaporated and the residue was dissolved in dichloromethane (50 mL), washed with 10% NaHCO_3_ solution (3 × 50 mL) and water (3 × 50 mL). The organic layer was concentrated under reduced pressure to afford crude products **1** or **2**.

Chemosensor **1** was synthesized starting from rhodamine B hydrazide (**4**) (1.3 g, 2.8 mmol), isophthaloyl dichloride (**3**) (0.29 g, 1.4 mmol) in THF (5 mL) and triethylamine (0.3 g, 3.0 mmol) in THF (70 mL). The crude product was recrystallized from a mixture of diethyl ether and dichloromethane (3:1) to afford the pure product (0.77 g, 52%) as a solid. mp 184 °C–186 °C. ^1^H NMR: δ 1.02–1.22 (m, 24H, CH_3_), 3.17–3.41 (m, 16H, CH_2_), 6.22–6.40 (m, 8H, ArH), 6.61–6.75 (m, 4H, ArH). 7.15 (d, *J* = 6.0 Hz, 2H, ArH), 7.39–7.47 (m, 2H, ArH), 7.47–7.56 (m, 4H, ArH), 7.61–7.69 (m, 2H, ArH), 7.96 (d, *J* = 6.0 Hz, 2H, ArH). ^13^C NMR: δ 12.6, 44.3, 97.7, 98.0, 107.9, 123.5, 124.2, 128.3, 129.3, 131.0, 133.1, 149.0, 153.7. ESI MS: *m/z* 1043.6, calculated: 1043.5 for [M+H]^+^.

Chemosensor **2** was prepared starting from rhodamine B hydrazide (**4**) (1.5 g, 3.3 mmol), benzene-1,3,5-tricarbonyl trichloride (**5**) (0.28 g, 1.1 mmol) in THF (5 mL) and triethylamine (0.35 g, 3.5 mmol) in THF (70 mL). The crude product was recrystallized from a mixture of diethyl ether and dichloromethane (3:1) to afford the pure product (1.02 g, 61%) as a solid. mp 204 °C–206 °C. ^1^H NMR: δ 1.05–1.24 (m, 36H, CH_3_), 3.19–3.39 (m, 24H, CH_2_), 6.21–6.41(m, 12H, ArH), 6.59–6.76 (m, 6H, ArH), 7.12 (d, *J* = 6.0 Hz, 3H, ArH), 7.38–7.56 (m, 6H, ArH), 7.88–8.01 (m, 6H, ArH). ^13^C NMR: δ 12.6, 44.2, 98.0, 99.9, 103.9, 107.9, 123.5, 124.2, 127.9, 129.2, 129.8, 132.9, 149.0, 153.6. ESI MS: *m/z* 1525.7, calculated: 1525.7 for [M+H]^+^.

### 3.4. Absorption and Fluorescence Measurements

Absorption and fluorescence titrations were performed in a 1 cm quartz cell by addition of small aliquots of metal ion work solutions to a 3 mL solution of proper amounts of **1** and **2** in CH_3_CN/H_2_O (3/1, *v*/*v*) and a 3 mL solution of a proper amount of **1** in CH_3_CN/HEPES buffer (0.02 M, pH = 7.0) (95/5, *v*/*v*). After thorough mixing, the solutions were allowed to stand at ambient temperature for 5 min, whereupon absorption or fluorescence spectra were recorded. Both the excitation and emission slits were 5 nm.

To determine the optimal conditions for Hg^2+^ detection, small aliquots of Hg^2+^, Cu^2+^, and Fe^3+^ work solutions were respectively added into 5 μM chemosensor **1** solutions which contained different fractions (5%, 10%, 15%, and 20%) of HEPES buffer (0.02 M, pH = 7.0) in CH_3_CN and mixed in a 1 cm quartz cell for 5 min. Then the fluorescence measurement was performed at ex/em = 510/580 nm.

## 4. Conclusions

In conclusion, the rhodamine derivatives **1** and **2** are very good fluorescent chemosensors, with a good selectivity toward Hg^2+^ and Fe^3+^ over other competitive ions in CH_3_CN/H_2_O (3/1, *v*/*v*). For practical Hg^2+^ detection, the experimental conditions were optimized to CH_3_CN/HEPES buffer (0.02 M, pH = 7.0) (95/5, *v*/*v*). Under these conditions, the fluorimetric quantification of Hg^2+^ by **1** was satisfactory in a linear working range of 0–50 μM, with a detection limit of 0.4 μM Hg^2+^ and a pH span of 4–10. These very good features make chemosensor **1** very promising for practical applications.

## Supplementary Information
