# Supplementary material for: Turn-On Fluorescent Chemosensor for Hg2+ Based on Multivalent Rhodamine Ligands
Source: Int J Mol Sci. 2012 Dec 7;13(12):16822–32. doi: 10.3390/ijms131216822 (PMC3546724; doi:10.3390/ijms131216822)
Supplement: Supplementary file 1 [file ijms-13-16822-s001.pdf]

Supplementary Information

Figure S1. Job's plot determination of (**a**) 1-Hg^{2+}, 1-Fe^{3+}, and (**b**) 2-Hg^{2+}, 2-Fe^{3+}. The total concentration of chemosensors and metal ions was kept at 0.1 mM.

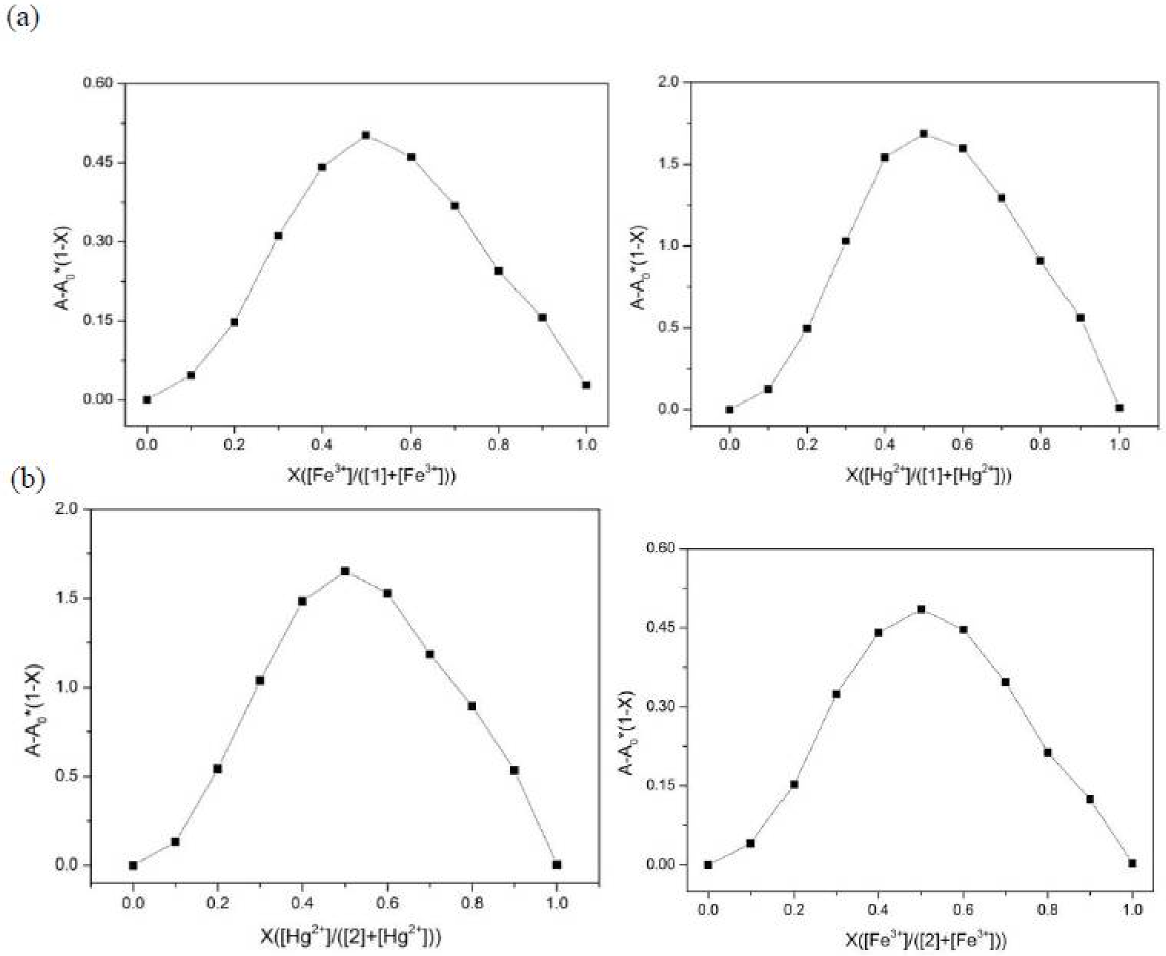

© 2012 by the authors; licensee MDPI, Basel, Switzerland. This article is an open access article distributed under the terms and conditions of the Creative Commons Attribution license (http://creativecommons.org/licenses/by/3.0/).
